# Deep learning–based acceleration of Compressed Sense MR imaging of the ankle

**DOI:** 10.1007/s00330-022-08919-9

**Published:** 2022-06-25

**Authors:** Sarah C. Foreman, Jan Neumann, Jessie Han, Norbert Harrasser, Kilian Weiss, Johannes M. Peeters, Dimitrios C. Karampinos, Marcus R. Makowski, Alexandra S. Gersing, Klaus Woertler

**Affiliations:** 1 Department of Radiology, Klinikum Rechts der Isar, Technische Universität München, Ismaninger Straße 22, 81675 Munich, Germany; 2 Department of Orthopaedic Surgery, Klinikum Rechts der Isar, Technische Universität München, Ismaninger Straße 22, 81675 Munich, Germany; 3 Philips GmbH, Röntgenstrasse 22, 22335 Hamburg, Germany; 4 Philips Healthcare, Veenpluis 4-6, Building QR-0.113, 5684 Best, PC Netherlands; 5 Department of Neuroradiology, University Hospital Munich (LMU), Marchioninistrasse 15, 81377 Munich, Germany

**Keywords:** Ankle, MRI, Deep learning, Compressed Sense

## Abstract

**Objectives:**

To evaluate a compressed sensing artificial intelligence framework (CSAI) to accelerate MRI acquisition of the ankle.

**Methods:**

Thirty patients were scanned at 3T. Axial T2-w, coronal T1-w, and coronal/sagittal intermediate-w scans with fat saturation were acquired using compressed sensing only (12:44 min, CS), CSAI with an acceleration factor of 4.6–5.3 (6:45 min, CSAI2x), and CSAI with an acceleration factor of 6.9–7.7 (4:46 min, CSAI3x). Moreover, a high-resolution axial T2-w scan was obtained using CSAI with a similar scan duration compared to CS. Depiction and presence of abnormalities were graded. Signal-to-noise and contrast-to-noise were calculated. Wilcoxon signed-rank test and Cohen’s kappa were used to compare CSAI with CS sequences.

**Results:**

The correlation was perfect between CS and CSAI2x (*κ* = 1.0) and excellent for CS and CSAI3x (*κ* = 0.86–1.0). No significant differences were found for the depiction of structures between CS and CSAI2x and the same abnormalities were detected in both protocols. For CSAI3x the depiction was graded lower (*p* ≤ 0.001), though most abnormalities were also detected. For CSAI2x contrast-to-noise fluid/muscle was higher compared to CS (*p* ≤ 0.05), while no differences were found for other tissues. Signal-to-noise and contrast-to-noise were higher for CSAI3x compared to CS (*p* ≤ 0.05). The high - resolution axial T2-w sequence specifically improved the depiction of tendons and the tibial nerve (*p* ≤ 0.005).

**Conclusions:**

Acquisition times can be reduced by 47% using CSAI compared to CS without decreasing diagnostic image quality. Reducing acquisition times by 63% is feasible but should be reserved for specific patients. The depiction of specific structures is improved using a high-resolution axial T2-w CSAI scan.

**Key Points:**

*• Prospective study showed that CSAI enables reduction in acquisition times by 47% without decreasing diagnostic image quality.*

*• Reducing acquisition times by 63% still produces images with an acceptable diagnostic accuracy but should be reserved for specific patients.*

*• CSAI may be implemented to scan at a higher resolution compared to standard CS images without increasing acquisition times.*

## Introduction

The ankle is one of the most complex joints with multiple oblique oriented ligaments and tendons [1–6]. In the United States, approximately 10,000 people suffer an ankle injury per day, the most frequent type of injury being sprains [7, 8]. Magnetic resonance (MR) imaging is one of the main imaging modalities for the assessment of musculoskeletal disorders on account of its high soft tissue contrast. However, the data acquisition process is inherently slow due to long encoding times [9]. This leads to long scan times and subsequently increased exam costs and reduced patient throughput. Moreover, the image quality is frequently compromised by motion artifacts, since remaining motionless for several minutes is a challenge even for healthy subjects [10].

Developments such as parallel imaging (PI) and, later, compressed sensing (CS) have accelerated MR image acquisition. CS reduces the number of acquired lines in k-space and restores the missing data through an iterative reconstruction algorithm [11, 12]. The combination of CS and PI was shown to reduce MR image acquisition times of the ankle by 20% without compromising diagnostic performance [13, 14]. More recently deep learning–based methods such as convolutional neural networks (CNNs) and generative adversarial networks (GAN) have shown promising results to accelerate the MR imaging data acquisition process [15]. These methods apply deep learning–based reconstruction schemes to create high-quality images from undersampled MR data [9, 16–21].

The aim of this study was to conduct a validation study to evaluate a compressed sensing artificial intelligence framework (CSAI) combining PI, CS, and deep learning–based artificial intelligence, for additional twofold and threefold acceleration of multi-contrast and multi-planar ankle MR imaging compared to conventional CS imaging and to compare the image quality and diagnostic performance between both techniques. In a secondary analysis, we compared the depiction of anatomical structures using high-resolution images reconstructed with CSAI.

## Materials and methods

### Subject selection

Thirty patients were prospectively enrolled in our study between January and June 2021 (15 female, age 19–84 years). Informed consent was obtained from all participants; the study was approved by the local institutional review board (42/21S). All patients were referred by the orthopedic department with various disorders including trauma, degeneration, and unclear pain. Individuals with conditions excluded by MR safety guidelines such as pacemakers, other implanted electronic devices or pregnancy, were not included.

### Data acquisition

In this study, the utility of a novel CNN that integrates and enhances the conventional CS algorithm referred to as Adaptive-CS-Network as presented by Pezzotti et al [15] was investigated (CSAI). The Adaptive-CS-Network mimics the iterative shrinkage–thresholding algorithm (ISTA) approach presented by Zhang et al [22] and integrates multiscale sparsification in a problem-specific learnable manner and combines a CNN-based sparsifying approach with the image reconstruction approach of compressed sense, which ensures data consistency and incorporates domain-specific prior knowledge such as coil sensitivity distribution and location of the image background. In this regard the Adaptive-CS-Network basically replaces the wavelet transform by a CNN as sparsifying transform in the compressed sense algorithm, still keeping domain-specific knowledge and a term ensuring data consistency in place in the reconstruction process. In contrast to the network presented by Pezzotti et al [15], the Adaptive-CS-Network employed in this work was pre-trained on about 740,000 sparsifying MR images using both 1.5T and 3T images of various anatomies and contrasts. Furthermore, the algorithm was optimized to allow execution on standard reconstruction hardware.

All examinations were performed on a 3T MR scanner (Ingenia Elition; Philips Healthcare) using a 16-channel ankle coil. The ankles were fixated within the coil to reduce motion artifacts. The examination protocol included the following 2D sequences: axial T2-weighted turbo spin echo (TSE) sequences, coronal T1-weighted TSE sequences and coronal as well as sagittal intermediate-weighted (IM) TSE sequences with spectral presaturation with inversion recovery (SPIR) for fat saturation. All sequences were obtained three times: (1) with an acceleration factor of 2.5 reconstructed using CS, (2) with an acceleration factor of 4.6–5.3 reconstructed using CSAI (CSAI2x), and (3) with an acceleration factor of 6.9–7.7 reconstructed using CSAI (CSAI3x). In addition, we obtained a high-resolution axial T2-weighted sequence reconstructed using CSAI (CSAIHR), with a similar scan duration but increased in-plane resolution and smaller slice thickness compared to the axial T2-weighted reference sequence reconstructed using CS only. To assess signal-to-noise (SNR) and contrast-to-noise (CNR), coronal and axial MR sequences were performed twice in 8 patients on the same ankle without repositioning between scans. The total scan duration of the CS protocol was 12:44 min. The scan duration of the CSAI2x protocol was 6:45 min (47% shorter compared to CS). The scan duration of the CSAI3x protocol was 4:46 min (63% shorter compared to CS). The reconstruction times of a multi-slice 2D scan were in the order of about 2 min. Further details of the imaging protocols are shown in Table 1.

**Table 1:** Sequence parameters of the sequences acquired using CS, CSAI2x, and CSAI 3x

Pulse sequence	ax T2w TSE	ax T2w TSE HR	cor T1w TSE	cor IMw TSE	sag IMw TSE
TR [ms]	2791	4733	705	2288	3631
TE [ms]	80	80	18	50	50
Echo train length (ETL)	15	15	6	15	8
Acquired resolution [mm]	0.3 × 0.5	0.2 × 0.3	0.3 × 0.4	0.4 × 0.6	0.4 × 0.5
Slice thickness [mm]	3	2	3	3	3
Field of view [mm]	130 × 130	130 × 130	140 × 140	140 × 140	140 × 140
Number of slices	31	46	23	23	24
Acceleration factor/scan time [min]					
CS	2.5/2:25	-	2.5/3:04	2.5/2:54	2.5/2:47
CSAI2x	5.1/1:30	5/2:50	5.2/1:44	5.2/1:45	4.6/1:34
CSAI3x	7.7/1:04	-	7.5/1:15	7.6/1:13	6.9/1:05

### Quantitative image analysis

The subtraction method was used to determine SNR and CNR values for CS, CSAI2x, and CSAI3x, as described previously [13, 23, 24]. Sequences acquired twice in the same exam session were subtracted to create noise maps. Regions of interest (ROIs) were placed in the same location on three consecutive slices in each series and the noise maps. SNR was calculated for the following tissues: fluid, muscle, and tendon. CNR was calculated for fluid/muscle, fluid/tendon, and muscle/tendon.

### Semi-quantitative image analysis

CS, CSAI2x, CSAI3x datasets, and CSAIHR images were analyzed in a randomized order with an interval of 4 weeks between readings to prevent recall bias. MR images were analyzed separately by two radiologists (25 and 7 years of experience; K.W. and S.C.F.), blinded to all clinical and other information. Readers graded depiction and presence of abnormalities of the tibiofibular syndesmosis, the medial and lateral ligament complex, the sinus tarsi ligaments, the extensor, flexor and peroneal tendons, the articular cartilage of the ankle joint, the bone, and the tibial nerve. Depiction of anatomical structures was graded using an ordinal 5-point Likert scale (1 = poor, 2 = below average, 3 = fair, 4 = good, 5 = excellent) evaluating the following criteria: partial volume effect, blurring, discrimination from adjacent structures, and signal homogeneity [23]. Abnormalities of the ligaments were graded in analogy to the Schweitzer classification system [25] as 0, no abnormality; 1, degenerative changes; 2, partial tear; 3, complete tear. Abnormalities of the tendons were graded as present/absent. Degenerative changes of the articular cartilage were graded as 0, no abnormality; 1, abnormal signal; 2, surface defect; 3, osteochondral defect. Bone abnormalities were graded as 0, no abnormality; 1, bone marrow edema pattern (BMEP); and 3, other abnormalities. Diagnostic confidence was recorded for all detected abnormalities with a 5-point ordinal scale as applied previously (1 = not detectable; 5 = 100% depicted, sharp) [23].

### Statistical analysis

The statistical analysis was performed with SPSS, version 25.0 (IBM) using a two-sided 0.05 level of significance (S.C.F.). We also differentiated between *p *≤ 0.05, *p *≤ 0.005, and *p* ≤ 0.001, since previous studies have shown that using *p* values above 0.005 may lead to a lack of reproducibility of scientific findings [26]. CS was used as standard of reference for all statistical comparisons since this is currently the standard imaging technique in our clinical routine. The Wilcoxon signed-rank test was used to assess differences in image quality and detection and classification of abnormalities between CS and CSAI protocols. Interobserver correlation and intersequence correlation of detected abnormalities were determined using Cohen’s kappa.

## Results

### Quantitative image analysis

**Fig. 1:**
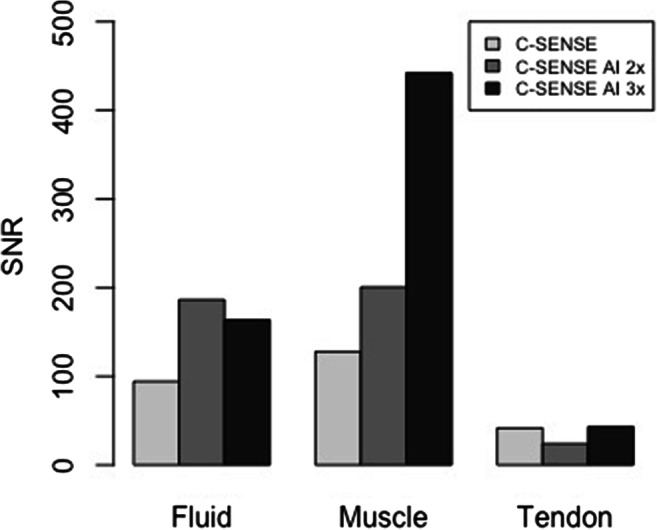
SNR of fluid, muscle, and tendons. No significant differences were found in SNR between the sequences acquired with CS only and the CSAI2x sequences. SNR was higher for fluid and muscle in the CSAI3x sequences compared to CS

**Fig. 2:**
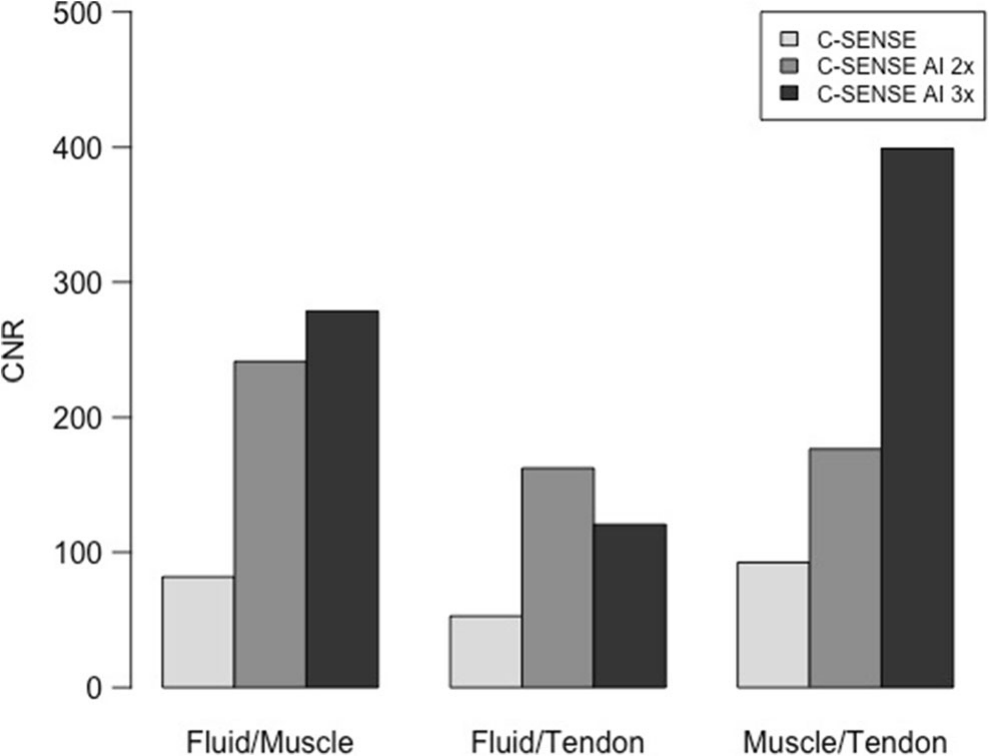
CNR calculated for the tissues fluid/muscle, fluid/tendon, and muscle/tendon

No significant differences were found in SNR between the CS and CSAI2x protocols. Compared to CS, SNR in the CSAI3x protocol was higher for fluid (94.2 ± 48.3 vs. 163.6 ± 77.7, *p* = 0.035) and muscle (127.8 ± 45.9 vs. 442.0 ± 17.3, *p* = 0.011); Fig. 1. Compared to CS, CNR in the CSAI2x protocol of fluid/muscle was higher (81.8 ± 37.6 vs. 241.1 ± 183.1, *p* = 0.035), while no significant differences were found for other investigated tissues. CNR was higher for all tissues in the CSAI3x protocol compared to CS only (fluid/muscle: 81.8 ± 37.6 vs. 278.4 ± 94.8, *p* = 0.011; fluid/tendon: 52.6 ± 37.7 vs. 120.6 ± 47.8, *p* = 0.011; muscle/tendon 92.4 ± 68.9 vs. 399.0 ± 49.2, *p* = 0.011; Fig. 2).

### Depiction of anatomical structures

**Fig. 3:**
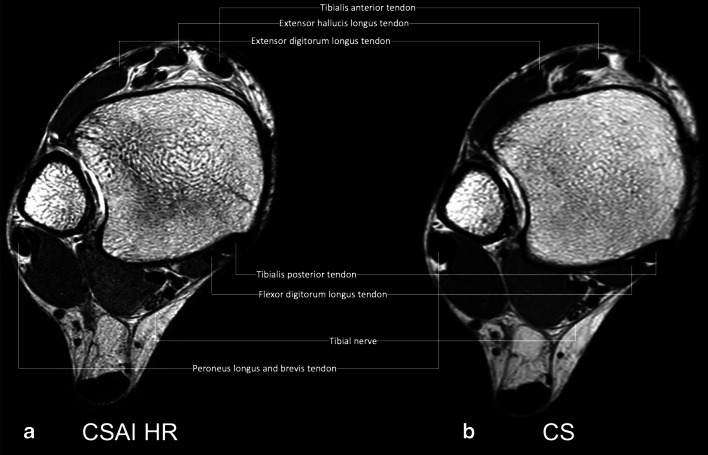
High-resolution axial T2-weighted CSAI TSE image (**a**, 2:50 min) and conventional CS TSE image (**b**, 2:25 min) of a 30-year-old female patient. Note the detailed depiction of the tendons and the tibial nerve and the improved discrimination from adjacent structures in the high-resolution CSAI TSE image compared to the CS TSE image

No significant differences were found for the depiction of all structures when comparing CS and CSAI2x. In CSAI3x the depiction was graded significantly lower (*p* ≤ 0.001) compared to CS for all anatomical structures apart from the tibionavicular ligament, which already had a low Likert score in CS and only marginally decreased in CSAI3x. Both readers found the high-resolution axial T2-weighted CSAIHR scan to improve the depiction of the peroneal tendon and the tibial bone structure (*p* ≤ 0.05, respectively), the anterior fibulotalar ligament and the extensor tendon (*p* ≤ 0.005, respectively), and the tibial nerve (*p* ≤ 0.001) (Fig. 3). In addition, reader 1 also graded the posterior tibiofibular ligament to be better depicted, reader 2 graded the flexor tendons, and the bone structure of the talus and fibula to be significantly better depicted (*p* ≤ 0.05, respectively). Detailed information on all graded anatomical structures is shown in Table 2. Magic angle artifacts were observed in all cases for CS, CSAI2x, CSAI3x, and CSAIHR. Moreover, no difference was noted for the markedness of the magic angle artifacts between different MR protocols.

**Table 2:** Depiction of anatomical structures

Anatomical complex	Reader 1				Reader 2			
	CS	CSAI 2x	CSAI 3x	CSAI HR	CS	CSAI 2x	CSAI 3x	CSAI HR
Tibiofibular syndesmosis								
Anterior tibiofibular ligament	4.1 ± 0.5	4.0 ± 0.6	3.5 ± 0.6***	4.2 ± 0.5	4.3 ± 0.6	4.1 ± 0.7	3.4 ± 0.6***	4.2 ± 0.5
Posterior tibiofibular ligament	4.6 ± 0.5	4.5 ± 0.5	3.9 ± 0.5***	4.8 ± 0.4*	4.7 ± 0.5	4.5 ± 0.7	4.0 ± 0.7***	4.8 ± 0.4
Lateral collateral ligament complex								
Anterior fibulotalar ligament	4.3 ± 0.6	4.2 ± 0.7	3.5 ± 0.7***	4.6 ± 0.6**	4.3 ± 0.6	4.3 ± 0.7	3.4 ± 0.7***	4.7 ± 0.5**
Calcaneofibular ligament	4.4 ± 0.5	4.3 ± 0.5	3.5 ± 0.7***	4.1 ± 0.7	4.1 ± 0.6	4.1 ± 0.6	3.4 ± 0.7***	4.2 ± 0.7
Posterior fibulotalar ligament	4.5 ± 0.5	4.4 ± 0.6	3.7 ± 0.6***	4.4 ± 0.5	4.5 ± 0.6	4.4 ± 0.6	3.6 ± 0.6***	4.4 ± 0.5
Medial collateral ligament complex								
Anterior tibiotalar ligament	3.7 ± 0.9	3.6 ± 0.9	3.2 ± 1.0***	3.8 ± 0.6	3.7 ± 1.0	3.7 ± 1.0	3.3 ± 1.0***	4.0 ± 0.7
Tibionavicular ligament	2.2 ± 1.2	2.1 ± 1.2	2.0 ± 1.2	n/a	2.1 ± 1.1	2.0 ± 1.1	2.0 ± 1.2	n/a
Tibiospring ligament	4.5 ± 0.5	4.5 ± 0.5	3.7 ± 0.4***	n/a	4.3 ± 0.7	4.3 ± 0.7	3.8 ± 0.6***	n/a
Tibiocalcaneal ligament	4.5 ± 0.6	4.6 ± 0.6	4.0 ± 0.4***	n/a	4.5 ± 0.6	4.6 ± 0.6	3.9 ± 0.5***	n/a
Posterior tibiotalar ligament	4.3 ± 0.5	4.6 ± 0.5	3.7 ± 0.5***	4.6 ± 0.5	4.7 ± 0.5	4.5 ± 0.6	3.7 ± 0.6***	4.7 ± 0.5
Ligaments within the sinus tarsi	4.3 ± 0.5	4.3 ± 0.5	3.8 ± 0.6***	n/a	4.4 ± 0.5	4.4 ± 0.6	3.8 ± 0.7***	n/a
Tendons								
Extensor tendons	4.5 ± 0.6	4.4 ± 0.6	3.8 ± 0.4***	5.0 ± 0.2**	4.5 ± 0.6	4.4 ± 0.6	4.1 ± 0.2***	4.9 ± 0.3**
Peroneal tendons	4.7 ± 0.4	4.7 ± 0.4	3.9 ± 0.5***	4.9 ± 0.3*	4.7 ± 0.5	4.7 ± 0.5	4.0 ± 0.5***	4.9 ± 0.3*
Flexor tendons	4.9 ± 0.3	4.9 ± 0.3	4.0 ± 0.4***	5.0 ± 0.0	4.9 ± 0.3	4.8 ± 0.4	4.1 ± 0.2***	5.0 ± 0.0*
Cartilage								
Fibulotalar cartilage	3.3 ± 0.6	3.0 ± 0.8	2.4 ± 0.7***	3.4 ± 0.8	3.3 ± 0.6	3.2 ± 0.8	2.5 ± 0.6***	3.5 ± 0.9
Tibiotalar cartilage	3.3 ± 0.5	3.2 ± 0.6	2.3 ± 0.5***	n/a	3.3 ± 0.6	3.3 ± 0.7	2.4 ± 0.6***	n/a
Bone								
Talus	4.9 ± 0.3	4.8 ± 0.4	3.5 ± 0.6***	5.0 ± 0.2	4.9 ± 0.3	4.8 ± 0.4	3.5 ± 0.6***	5.0 ± 0.0*
Fibula	4.9 ± 0.3	4.9 ± 0.3	3.7 ± 0.6***	5.0 ± 0.0	4.8 ± 0.4	4.8 ± 0.4	3.8 ± 0.6***	5.0 ± 0.0*
Tibia	4.9 ± 0.3	4.8 ± 0.4	3.7 ± 0.6***	5.0 ± 0.0*	4.8 ± 0.4	4.7 ± 0.5	3.8 ± 0.6***	5.0 ± 0.0*
Tibial Nerve	4.0 ± 0.5	4.0 ± 0.5	3.2 ± 0.7***	4.8 ± 0.4***	4.0 ± 0.5	4.0 ± 0.6	3.2 ± 0.7***	4.9 ± 0.3***

Data are presented as means ± standard deviations

5-point Likert scale (5 = best; 1 = worst)

*p ≤ 0.05 using CS as standard of reference

**p ≤ 0.005 using CS as standard of reference

***p ≤ 0.001 using CS as standard of reference

### Assessment of abnormalities

**Fig. 4:**
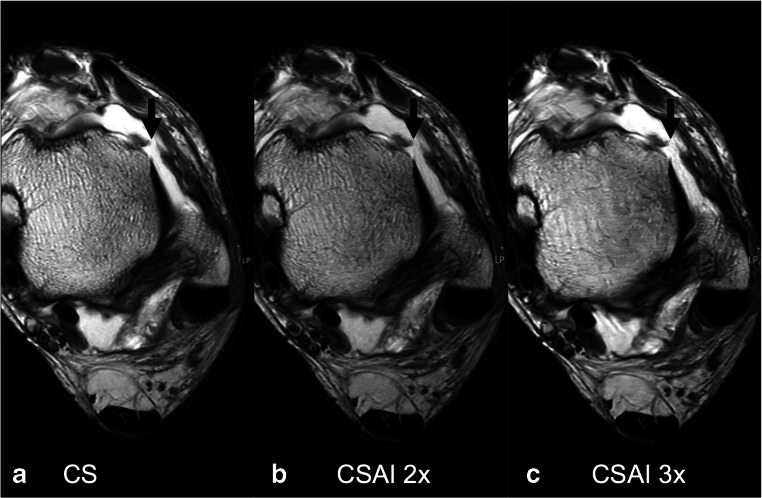
Axial T2-weighted TSE images of a 52-year-old male acquired using CS (**a**), CSAI2x (**b**), and CSAI3x (**c**), approximately reducing the acquisition time by half and two-thirds compared to CS, respectively. The images show a complete tear of the anterior talofibular ligament (black arrow). Note the slightly increased blurriness of the tibia on the CSAI3x images. However, the tear of the anterior talofibular ligament is clearly depicted on all three images

Details of the assessment of abnormalities and the diagnostic confidence are given in Table 3. All abnormalities detected on images reconstructed with CS were also detected on CSAI2x images, and there was no significant difference in the diagnostic confidence recorded for all detected abnormalities between both protocols (Fig. 4). Moreover, most abnormalities detected on images obtained with CS were also detected on CSAI3x images (*n *= 47/49, reader 1; *n* = 44/45, reader 2). Only one longitudinal split tear of the peroneus brevis tendon and one bone abnormality (bone marrow edema of the talus, < 3 mm diameter) recorded on images acquired using CS were not seen in CSAI3x images, due to increased blurring of the anatomical structures. Both readers recorded a significantly lower diagnostic confidence for abnormalities of the lateral ligament complex and the bone on CSAI3x compared to CS images (*p* ≤ 0.005). Reader 1 also recorded a lower diagnostic confidence for abnormalities of the cartilage, while reader 2 recorded a lower diagnostic confidence for tendon abnormalities on CSAI3x compared to CS images (*p* ≤ 0.05). No new, artificial abnormalities were created by CSAI reconstructions.

**Table 3:** Detected abnormalities and diagnostic confidence

Anatomical complex	Grade	CS		CSAI 2x		CSAI 3x	
		*n*	Rating	*n*	Rating	*n*	Rating
Reader I							
Tibiofibular syndesmosis	I						
	II	1		1		1	
	III						
Total		1	4.0 ± 0.0	1	4.0 ± 0.0	1	4.0 ± 0.0
Lateral collateral ligament complex	I	5		5		5	
	II	5		5		6	
	III	5		5		4	
Total		15	4.5 ± 0.5	15	4.4 ± 0.5	15	3.9 ± 0.5**
Medial collateral ligament complex	I	2		2		2	
	II						
	III						
Total		2	4.0 ± 0.0	2	4.0 ± 0.0	2	3.0 ± 0.0
Tendons		13	4.6 ± 0.5	13	4.5 ± 0.5	12	4.1 ± 0.5
Cartilage	I						
	II						
	III	4		4		4	
Total		4	5.0 ± 0.0	4	4.8 ± 0.5	4	4.0 ± 0.0*
Bone	I	7		7		6	
	II	7		7		7	
Total		14	5.0 ± 0.0	14	5.0 ± 0.0	13	4.3 ± 0.5**
Reader II							
Tibiofibular syndesmosis	I						
	II	1		1		1	
	III						
Total		1	4.0 ± 0.0	1	4.0 ± 0.0	1	4.0 ± 0.0
Lateral collateral ligament complex	I	4		4		4	
	II	5		5		6	
	III	5		5		4	
Total		14	4.3 ± 0.5	14	4.2 ± 0.6	14	3.8 ± 0.4**
Medial collateral ligament complex	I	1		1		1	
	II						
	III						
Total		1	4.0 ± 0.0	1	4.0 ± 0.0	1	3.0 ± 0.0
Tendons		11	4.5 ± 0.5	11	4.3 ± 0.5	11	3.7 ± 0.5*
Cartilage	I						
	II					1	
	III	4		4		3	
Total		4	4.5 ± 0.6	4	4.5 ± 0.6	4	4.0 ± 0.0
Bone	I	7		7		6	
	II	7		7		7	
Total		14	5.0 ± 0.0	14	5.0 ± 0.0	13	4.2 ± 0.4**

Data are presented as means ± standard deviations

5-point Likert scale (5 = best; 1 = worst)

*p ≤ 0.05 using CS as standard of reference

**p ≤ 0.005 using CS as standard of reference

**Fig. 5:**
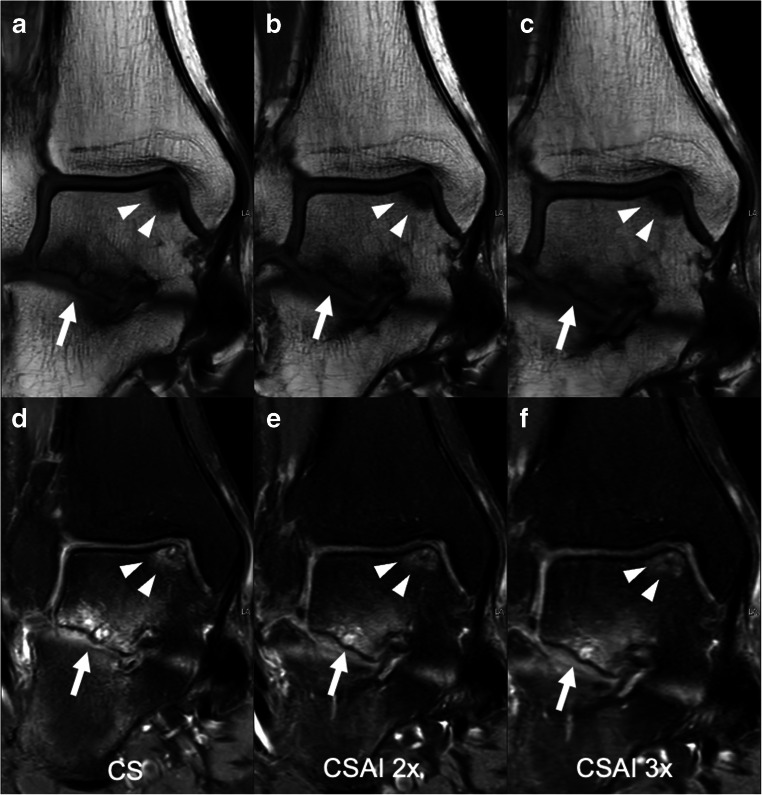
Coronal T1-weighted and intermediate-weighted TSE images with spectral presaturation with inversion recovery (SPIR) for fat saturation of a 42-year-old male, acquired using CS (**a**, **d**), CSAI2x (**b**, **e**), and CSAI3x (**c**, **f**) showing an osteochondral defect of the medial talar shoulder (white arrowheads) as well as degenerative changes of the subtalar joint (white arrow). The depiction of bone was rated equally on CS and CSAI2x images with a Likert score of 5 (excellent), but slightly lower on CSAI3x images with a Likert score of 4 (good)

Interreader correlation was excellent for all criteria with *κ* = 0.86–1.0. Intersequence correlation was perfect for CSAI2x and CS for both readers with *κ* = 1.0, respectively. Intersequence correlation was excellent for CSAI3x and CS for both readers with *κ* = 0.93–1.0 for reader 1 and *κ* = 0.86–1.0 for reader 2, respectively (Fig. 5).

## Discussion

In this study, we implemented an artificial intelligence framework for additional twofold and threefold acceleration of ankle MR imaging compared to conventional CS. The correlation was perfect for CS and CSAI2x and excellent for CS and CSAI3x. No significant differences were found for the depiction of anatomical structures between CS and CSAI2x and the same abnormalities were detected in both sequences. For CSAI3x the depiction was graded significantly lower compared to CS; however, most abnormalities were also detected. The high-resolution axial T2-weighted sequence was found to specifically improve the depiction of tendons, bone and the tibial nerve, with only minimally increased scan time compared to the axial T2-weighted CS reference scan.

Previous studies have used deep learning to accelerate MR imaging. One method is to create high-resolution from low-resolution data [27–29]. Chaudhari et al used deep learning CNNs to generate thin-slice knee MR images from thicker input slices [27]. Moreover, different deep learning methods have been applied to reconstruct MR images from undersampled MR data [17, 30]. Akçakaya et al used a k-space-based deep learning technique to estimate missing k-space lines from acquired k-space data [17]. Liu et al used a generative adversarial network (GAN) to enforce data consistency for a robust reconstruction of accelerated MR images [30]. In a similar approach to our study, Hammernik et al used an image-space-based technique for reconstruction of accelerated MR images with a so-called variational network [16]. The overall image quality of the variational network reconstructions with an acceleration factor of 4 was graded equal or better compared to conventional PI compressed sensing images, though anatomical structures and pathologies were not separately evaluated [16]. In accordance, we found no significant differences for the depiction of anatomical structures between CS and CSAI2x with an acceleration factor of 4.6–5.3 equating to approximately half the acquisition time of CS only. Moreover, the same abnormalities were detected in both sequences with no significant difference in the recorded diagnostic confidence. These findings suggest that CSAI2x protocols could be implemented in clinical practice without decreasing diagnostic image quality, though further validation studies are warranted to confirm these results.

In addition, we also evaluated the diagnostic image quality for CSAI3x with a threefold acceleration of acquisition times compared to the CS protocol. The depiction and diagnostic confidence for the detection of abnormalities was graded lower for most structures compared to CS. The reduction in diagnostic image quality was caused by increased image blurring, resulting in a reduced sensitivity in the detection of abnormalities. No spurious artifacts, which overlaid or mimicked a pathology, have been observed. However, it should be noted that only one peroneal tendon disorder and one bone abnormality of the talus recorded in CS were not seen in CSAI3x, while all other abnormalities were detected. These findings suggest that CSAI3x could be used in specific cases when the patient is in significant pain or only able to remain motionless for a short time.

Measured values of SNR were higher in CSAI3x, but not in CSAI2x compared to CS. For CSAI3x the higher SNR values were offset by image blurring and a related, significantly worse depiction of anatomical structures and abnormalities. This indicates that SNR and CNR values do not represent the total perceived image quality, when comparing iterative image reconstructions and image denoising. While image details and abnormalities were depicted worse in CSAI3x, no artificial details or abnormalities were observed.

Despite promising results, some limitations are pertinent to this study that should be addressed in further studies. No external standard of reference, such as arthroscopy, was available to serve as ground truth for detected pathologies of the ankle. Therefore, it was not possible to verify the diagnosed pathologies using an external standard of reference. Moreover, our series included only 30 subjects with a limited number of different pathologies and, thus, further studies including more patients are warranted to confirm these results. The aim of this study was to conduct a validation study on an internal data set. However, further studies are warranted to validate these results. Next steps include temporal validation (evaluation on a second data set from the same center) and external validation (evaluation on data from one or more other centers) [31]. To implement CSAI in the clinical workflow, it may also be beneficial to initially introduce these imaging sequences in addition to standard routine imaging protocols. This approach would allow for radiologists to develop a better understanding of CSAI sequences and when to best implement them.

In conclusion, we conducted a validation study from a single institution to evaluate a compressed sensing artificial intelligence framework and found that acquisition times can be reduced by 47% using CSAI without decreasing diagnostic image quality. Reducing acquisition times by 63% still produces images with an acceptable diagnostic accuracy but should be reserved for specific cases when patients are in significant pain or only able to remain motionless for a short time. This study is the foundation for further validation studies.

## Abbreviations

BMEP: Bone marrow edema pattern
CNN: Convolutional neural network
CNR: Contrast-to-noise ratio
CS: Compressed sensing
CSAI: Compressed sensing artificial intelligence
CSAI2x: CSAI with an acceleration factor of 4.6–5.3
CSAI3x: CSAI with an acceleration factor of 6.9–7.7
CSAIHR: CSAI high-resolution axial T2-weighted sequence
ETL: Echo train length
GAN: Generative adversarial network
IM: Intermediate-weighted
ISTA: Iterative shrinkage–thresholding algorithm
MR: Magnetic resonance
PI: Parallel imaging
ROI: Region of interest
SNR: Signal-to-noise-ratio
SPIR: Spectral presaturation with inversion recovery
TSE: Turbo-spin-echo

## Declarations

### Guarantor

The scientific guarantor of this publication is Klaus Woertler.

### Statistics and biometry

One of the authors has significant statistical expertise: Sarah C. Foreman.

### Methodology

• prospective

• cross sectional study

• performed at one institution
